# Closure of the Abdominal Incision after Laporotomy and the Tendency to Hernia

**Published:** 1899-08

**Authors:** 


### Abstracts and Selections.

### Closure of the Abdominal Incision After Laporotomy and the Tendency to Hernia.

In the course of time, abdominal operators have reached a proficiency in technique and an assurance in the application of the details of asepsis that have made laparotomy a comparatively facile and safe procedure. There has, however, remained an objection not foreseen at first, but ever becoming more insistently prominent as the number of abdominal operations increased. Despite the most anxious care and most solicitous technique, ventral herniæ occur at the site of the abdominal incision and often make life miserable for the patient. The frequency of the occurrence of hernia has become one of the great sources of opprobrium to modern abdominal surgery, and it is not unusual to have patients who do not fear the result of the operation itself hesitate to undergo it because of the fear of the subsequent hernia that they have learned to dread from the experience of friends or acquaintances.

The review of the recent results of post-operational hernia, by Dr. John G. Clark, of Johns Hopkins Hospital, in a recent number of *Progressive Medicine*,* shows that a number of factors which have usually been considered as influencing the production of hernia really have no aetiological connection with it. For instance, permitting the patients to get up after seventeen or eighteen days does not predispose to hernia, and keeping them in bed for longer periods does not prove a prophylactic against its occurrence. The wearing or failure to wear a bandage after operation does not affect the liability to hernia, either favorably or unfavorably. Pregnancy following immediately or remotely after operation plays no part in the production of hernia despite preconceived notions to the contrary.

It is evident then that the occurrence of ventral hernia after oper-

*Progressive Medicine. A Quarterly Digest of Advances, Discoveries and Improvements in the Medical and Surgical Sciences. Edited by Hobart Amory Hare, M. D. Volume II. June, 1899. Lea Brothers & Co., Philadelphia.

ation is mainly due to the method of closing the abdominal wound, despite all that has been said by certain gynecologists abroad as to the advantage to be derived in this matter from making the incision through the rectus muscle. Dr. Clark, from his experience at Johns Hopkins as well as his records of the subject, decides in favor of the incision in the linea alba. Two things are necessary to lessen the tendency to hernia in closing the incision. First, the fascia, *i. e.,* the aponeurosis of the recti muscles, must be carefully brought together so as to secure complete and firm continuous union along the line of section. The essential point in placing the sutures is to catch enough of the aponeurosis to firmly bring the borders of the fascia not only into complete coaptation, but also to slightly elevate them into a median ridge. The coaptation of the fascia must be especially exact at the lower end of the incision when the liability to hernia is greater, because the layers of fascia are fewer.

The second requisite for a firm cicatrix is to secure healing *per primam,* and this is best secured by leaving no dead spaces in which blood or lymph may collect to become infected, and by allowing no penetrating cutaneous stitches through which micro-organisms may penetrate from the surface despite the most careful precautions. On the whole, this subject of the avoidance of hernia by a careful technique in the closure of the abdominal incision would seem to have reached a development that leaves very little to be desired, and it is evident that it is only in patients with especially relaxed tissues or with natural tendencies to hernia that the operator may feel exempt from responsibility in future cases of this annoying sequela.

